# Clinical response in dogs with acute hemorrhagic diarrhea syndrome following randomized probiotic treatment or fecal microbiota transplant

**DOI:** 10.3389/fvets.2023.1050538

**Published:** 2023-02-02

**Authors:** Maria C. Jugan, Kate KuKanich, Leah Freilich

**Affiliations:** Department of Clinical Sciences, College of Veterinary Medicine, Kansas State University, Manhattan, KS, United States

**Keywords:** bacterial translocation, endotoxemia, gastrointestinal permeability, hemorrhagic gastroenteritis, microbiome

## Abstract

Probiotics and fecal microbiota transplants (FMTs) are two microbiome-targeted therapies that have been investigated for use in gastrointestinal diseases associated with dysbiosis. The aim of this study was to compare the effects of an oral multi-strain probiotic and enema-administered FMTs on clinical signs and serum lipopolysaccharide in dogs with acute hemorrhagic diarrhea syndrome (AHDS). A total of 18 client-owned dogs with a diagnosis of AHDS were enrolled in a randomized, blinded study at the time of hospital admission. The dogs were randomized into two groups: the probiotic group received a daily oral probiotic (200 × 10^9^ CFU/10kg q 24 h) for 14 days and a single sham enema; the FMT group received a single FMT *via* retention enema (10 mL/kg) and placebo oral capsule for 14 days. All dogs received concurrent standard-of-care therapy, including intravenous fluids and anti-emetics; no dogs received antimicrobials. The fecal score, disease severity scores, and serum lipopolysaccharide were measured on days 0, 3, and 14. Fourteen of eighteen enrolled dogs completed the study (*n* = 9 probiotics; *n* = 5 FMT). Lipopolysaccharide decreased on days 3 and 14 from baseline and correlated with fecal and disease severity scores. There was no difference in the duration or severity of clinical signs in dogs with AHDS following an enema-administered FMT compared to probiotic treatment. Further evaluation of serum lipopolysaccharide as a marker of disease severity and recovery is warranted.

## 1. Introduction

Acute hemorrhagic diarrhea syndrome (AHDS) is a common cause of severe, hemorrhagic diarrhea and vomiting in dogs associated with gastrointestinal (GI) bacterial dysbiosis (1–4) and hyperpermeability (2, 4). Previous studies have inferred associations with shifts in normal bacterial communities, increases in potentially pathogenic bacteria targeted for analysis (e.g., *Clostridium perfringens*) (2–5), endotoxin production (6–8), and dietary factors (1), but the presence of these predisposing factors is inconsistent in AHDS dogs (6–11) and in some cases, can be found in healthy dogs (e.g., fecal *Clostridium perfringens*) (8, 10). Therefore, AHDS is considered an idiopathic condition, with standard therapy consisting of intensive intravenous fluids and supportive care. However, crucial roles of the normal GI microbiome include maintaining the GI barrier and protecting GI mucosa from pathogenic species (12, 13), making microbiome-targeted therapies intriguing ancillary treatments. Serum lipopolysaccharide (LPS) is a molecule on the outer membrane of gram-negative bacteria.

Disruption of the normal microbiome, along with subsequently increased GI permeability, could allow the translocation of intraluminal GI bacteria into the bloodstream and result in increased measurable LPS concentrations. Through the potential to restore normobiosis and decrease GI hyperpermeability, microbiome-targeted therapies could improve clinical signs more rapidly and decrease hospitalization time.

Microbiome-targeted therapies have been investigated as the treatment for both acute and chronic GI diseases in humans and veterinary species. Treatments do not have equal efficacy among disease processes (14–17); differences in treatment responses apply to the type of treatment (e.g., probiotic vs. fecal microbiota transplant) and bacterial species contained within commercial products (18). In AHDS dogs, antimicrobials targeting an abnormal GI microbiome have not improved clinical outcomes vs. placebo treatment (19). Possible benefits of novel microbiome-targeted therapies, such as probiotics or fecal microbiota transplant (FMT), or which therapy provides the greatest benefit have not been fully explored in AHDS. Probiotic administration in AHDS dogs improved measures of GI microbiome dysbiosis vs. placebo (6). Furthermore, probiotic treatment might shorten the duration of acute, non-hemorrhagic diarrhea in dogs (20–22). FMT decreased diarrhea duration and hospitalization time in puppies with enteric parvovirus (23) and improved fecal consistency and dysbiosis measurements in dogs with acute, non-hemorrhagic diarrhea (24). In dogs with AHDS, FMT increased fecal bacterial communities responsible for short-chain fatty acid production, but there was no difference in clinical disease scores at the time of discharge between FMT-treated dogs vs. controls (25).

Although there have been preliminary investigations of FMT and probiotics in different AHDS populations, FMT and probiotics have not been directly compared. This study was designed to compare FMT vs. probiotic effects on duration and severity of clinical signs (i.e., hospitalization time and fecal scores) and serum LPS, as a marker of GI permeability, in a single population of AHDS dogs. A secondary objective was to determine whether serum LPS concentrations correlated with fecal scores or disease severity scores.

## 2. Materials and methods

### 2.1. Study population

This prospective, randomized single-site study enrolled client-owned dogs at the time of hospitalization for AHDS following informed owner consent. No direct financial incentive was provided to owners for study participation; although, costs of screening CBC and biochemistry profile, rechecking CBC, and 72-h and 14-day recheck examination fees were covered by the study. Procedures were approved by the Kansas State University IACUC (protocol 4237.1).

Inclusion criteria were defined as < 48 h duration hemorrhagic diarrhea and/or vomiting, Hct > 50% with normal serum total protein prior to treatments, including intravenous fluids, and exclusion of systemic illness based on CBC and serum biochemistry panel demonstrating the absence of clinically significant systemic disease (e.g., renal azotemia, hyperbilirubinemia, liver enzyme elevation >2x the reference interval); urine specific gravity (USG) was performed to confirm prerenal azotemia (USG ≥ 1.035), as needed. Dogs had a negative fecal flotation for GI parasites (centrifugal Sheather's sugar solution) and parvovirus antigen test (SNAP Parvo Test, IDEXX Laboratories, Westbrook, ME, USA). Additional tests, based on screening blood work (e.g., baseline cortisol) or abdominal imaging, were performed at the discretion of the attending clinician, and dogs with positive results were excluded. Dogs with severe disease, defined as total leukocyte count >18.0 × 10^9^/L or < 5.0 × 10^9^/L plus ≥ 1 additional systemic inflammatory response criteria (26) or persistent hypotension (< 90 mmHg systolic *via* Doppler) following fluid resuscitation, were excluded. Other exclusion criteria were chronic GI signs (either >48 h or history of intermittent, recurrent GI signs), treatment to control historical GI signs (e.g., novel protein diet), or administration of antimicrobials, steroids, or probiotics within the previous month, as well as other clinical signs or documented biochemical or imaging evidence of systemic, non-GI disease. Criteria for withdrawal and rescue antimicrobial administration included the development of new fever in-hospital or persistent fever (>39.7°C) > 8 h after admission, hypotension refractory to intravenous fluids, or development of neutropenia, degenerative left shift, or thrombocytopenia.

### 2.2. Fecal microbiota donors

Three healthy dogs were recruited prospectively from staff pets. Dogs were determined healthy based on normal physical examination, including a body condition score (BCS) 4–6/9 (27), absence of GI signs within the previous 6 months and lack of dietary management to control historical GI signs, no medications other than routine heartworm or flea prevention within the previous 4 months, and a normal CBC and serum biochemistry profile. Dogs had a negative fecal flotation, *Giardia* antigen ELISA (SNAP *Giardia* Test, IDEXX Laboratories, Westbrook, ME, USA; performed through the Kansas State Veterinary Diagnostic Laboratory), and fecal infectious disease PCR panel (*Cryptosporidium, Salmonella, C. perfringens* enterotoxin alpha gene, canine enteric coronavirus, canine parvovirus 2, canine distemper virus; Kansas State Veterinary Diagnostic Laboratory) within 2 weeks of fecal donation. No FMT donors received raw diets or raw treats. Three donors were enrolled to allow the utilization of fecal material within the desired storage time, with a subsequent donor enrolled after the maximum storage time was reached.

### 2.3. FMT preparation

Donor fecal samples were collected at the time of defecation, refrigerated, and prepared within 4 h. Sample preparation and storage were based on techniques described in human and veterinary literature (28, 29). Samples were blended with a ratio of 50 g fecal material to 250 mL sterile 0.9% saline. The mixture was sieved through sterile gauze to remove solid particles. The sieved material was resuspended to 50% of the original volume in sterile 0.9% saline. The solid material was sieved, and the remaining liquid was blended with 10% (volume:volume) >99% sterile glycerol. The mixture was frozen in 50 mL aliquots at −80°C for a maximum of 6 months, a period of time with demonstrated microbiome stability (30). Two hours prior to FMT, mixtures were thawed at room temperature. Each FMT mixture was from an individual donor (i.e., samples were not pooled).

### 2.4. Study design

**Figure 1:**
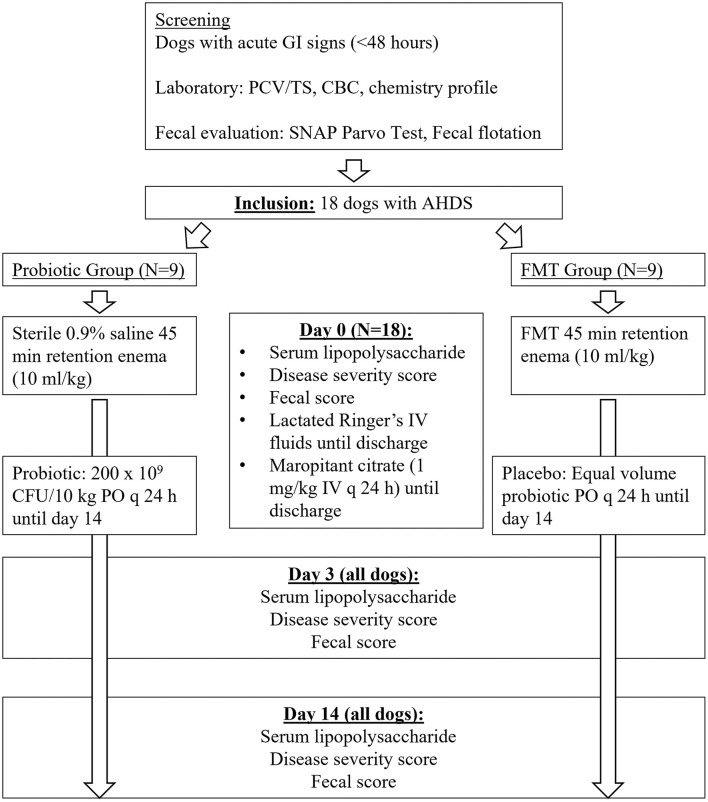
Study timeline for 18 dogs with acute hemorrhagic diarrhea syndrome receiving either fecal microbiota transplant (FMT) or probiotic.

As there was no available veterinary literature evaluating shifts in GI microbiota following FMT at the time of study design, *a priori* sample size calculation was based on a human dysbiosis model demonstrating 70% recovery of the normal GI microbiota following FMT vs. 15% with probiotic, (31) with the expectation that a normal fecal score would relate to dysbiosis resolution and estimated nine dogs per group for statistical significance. Figure 1 demonstrates the study timeline. Study dogs were divided into two treatment groups (probiotic or FMT) *via* simple randomization (coin-flip at the time of patient presentation), blocked into four groups of 4 and one group of 2 until nine dogs were included in each group. Each block consisted of an equal number of dogs in each treatment group. Enrolling dogs in blocks allowed efficient utilization of stored fecal material uniformly over the course of the study, accounting for maximum storage time. Study investigators (MJ, LF) played no role in patient admission and were contacted following patient hospitalization. Owners and investigators performing fecal scoring were blinded to the treatment group. Attending clinicians were not blinded to the treatment group; the investigator performing fecal scoring was not involved in patient treatment. At enrollment, owners completed a questionnaire detailing diet and treatment history, medications, supplements, dietary indiscretion, and historical medical conditions.

Probiotic dogs received a multi-strain probiotic (Visbiome, ExeGi Pharma, Rockville, MD, USA; 450 CFU/packet, *Streptococcus thermophiles, Bifidobacterium breve, B. longum, B. infantis, Lactobacillus acidophilus, L. plantarum, L. paracasei, L. delbrueckii bulgaricus*) at 200 × 10^9^ CFU/10kg q 24 h on food throughout the 14-day study (32). FMT dogs received a single FMT as a 45-min retention enema (10 mL/kg) within 8 h of admission. Placebo treatments were administered to all dogs. In dogs that did not readily ingest the probiotic or placebo during the initial hospitalization period, the products were mixed with water (6 mL) and administered as a slurry to ensure the full dose was received. Probiotic dogs received a sham FMT (sterile saline at 10 mL/kg) within 8 h of admission to account for possible effects of enema administration on stool consistency or the microbiome. FMT dogs received a daily oral placebo powder (maltodextrin; volume = probiotic volume/body weight) for study duration to maintain owner blinding and account for the placebo effect on owner assessment of clinical signs. Dogs were not walked or fed for 4 h post-enema. After discharge from the hospital, probiotic and placebo products were refrigerated by owners and administered on the dog's food once daily until study completion.

All dogs received intravenous fluids (lactated Ringer's solution), with the rate adjusted for the individual dog, and maropitant (Cerenia, Zoetis, Parsippany, NJ, USA; 1 mg/kg IV q 24 h) during hospitalization. All dogs received a standardized canned commercial diet (Purina Pro Plan Veterinary Diets EN Gastroenteric Canine Formula, Nestle Purina PetCare, St. Louis, MO, USA) in-hospital, which was fed at 1/4 resting energy requirement (RER) every 6 h, beginning 12 h after admission. Dogs were discharged with a recommendation to continue this diet through day 14. Recommended criteria for discharge included the resolution of vomiting, eating >75% RER, and improvement but not the resolution of diarrhea.

#### 2.4.1. Hematologic and biochemical analyses

Blood was drawn on admission (CBC, biochemistry panel, LPS), day 3 (LPS), and day 14 (LPS). Blood for CBC was collected into EDTA tubes. Blood for the biochemistry profile and LPS were collected into two separate plastic serum clot-activator vacutainer tubes. CBC and biochemistry profiles were analyzed at the time of sample collection through the Kansas State Veterinary Diagnostic Laboratory.

Blood for LPS was allowed to clot, centrifuged (20 min, 3,000 × g, 20°C), serum separated manually with pipettes, and frozen immediately or refrigerated overnight, and stored at −80°C. LPS concentrations were analyzed in bulk at study completion using a commercially available canine ELISA according to manufacturer instructions (MyBioSource, San Diego, CA, USA) (33, 34). The LPS assay used was validated with bovine serum albumin standards by the manufacturer. The capture antibody is a mouse monoclonal antibody. The detection antibody is a rabbit polyclonal antibody. Immunogen for both antibodies was OVA-LPS. Samples with gross hemolysis or lipemia underwent a second centrifugation cycle. Following a second centrifugation, only samples determined acceptable by visual examination were used. Samples were run in duplicate, with 2–3 sample pairs repeated on the same plate for intra-assay variability. Thirty-five sample pairs were repeated across three plates for inter-assay variability. Baseline LPS concentrations were compared between samples collected in the first 3 and 6 months of the study to samples collected in the last 3 and 6 months of the study, with no difference noted (*P* > 0.99).

#### 2.4.2. Fecal scoring

Fresh fecal samples were scored on days 0, 3, and 14 using a 1–5 scoring system (35) based on gross stool consistency by a single-blinded investigator (KK). A score of “1” represents feces that “crumble with little pressure,” and a score of “5” represents liquid diarrhea (35). Owners documented fecal scores daily following discharge to determine when dogs achieved a fecal score ≤ 3. Owners were given a picture-based scoring chart, and the dog's fecal score at the time of discharge was demonstrated for reference. A fecal score ≥3.5 was considered diarrhea.

#### 2.4.3. Disease severity scoring

Disease severity scoring was performed on days 0, 3, and 14 using a canine AHDS scoring system based on clinical signs, including appetite, vomiting frequency, stool consistency, defecation frequency, and estimated dehydration, with a higher cumulative score indicating more severe disease (19); the activity level was not included in the numerical score. This scoring system has been used in studies evaluating the clinical course of AHDS (6, 19, 25, 36).

### 2.5. Statistical analysis

Statistical analysis was performed using commercial software (GraphPad Prism 9.1.2, GraphPad Software, San Diego, CA, USA; IBM SPSS Statistics 8.0, IBM Corporation, Armonk, NY, USA) Data were assessed for normality using the Shapiro–Wilk test and reported as mean ± SD for normally distributed data or median (range) for nonnormally distributed data. Significance was set at *p* < 0.05. Analysis was performed on an intention-to-treat basis.

Baseline population characteristics, clinicopathologic values, fecal score, disease severity scores, LPS concentration, days to discharge, and days to fecal score 3 (i.e., normal stool consistency) were compared between groups using standardized differences (Cohen's *d*), where a value >0.25 was considered to indicate a difference between groups (37). Fecal scores and disease severity scores on days 0, 3, and 14 were compared using a mixed logistic regression model, accounting for repeated measures, as well as day and treatment effects. Serum LPS on days 0, 3, and 14 were compared using a mixed effects model to account for missing values using a compound symmetry covariance matrix and fit using restricted maximum likelihood; in the absence of missing values, this is equivalent to a mixed analysis of variance with repeated measures. Within-subject effects (time, interaction of treatment × time), between-subjects effects (treatment [i.e., probiotic]), and Geisser–Greenhouse adjustment were included. When the full model was significant, Sidak's test was used to compare each time point. Spearman's correlation (r_s_) was used to compare fecal score and disease severity score with LPS. The correlation was defined as previously described (0–0.09 = negligible, 0.1–0.39 = weak, 0.4–0.69 = moderate, 0.7–0.89 = strong, 0.9–1.0 = very strong) (38). Fisher's exact test was used to compare study completion rates between groups.

## 3. Results

### 3.1. Study population

Following AHDS diagnosis and exclusion of concurrent diseases, 18 client-owned dogs were enrolled from January 2020 to March 2021 with informed owner consent. The study population included five castrated males (*n* = 1 FMT; *n* = 4 probiotic), one intact male (FMT), eight spayed females (*n* = 4 FMT; *n* = 4 probiotic), and four intact females (*n* = 3 FMT; *n* = 1 probiotic). Breeds included three Labrador retrievers, three Pitbull Terriers, two German shepherds, two Chihuahuas, two Shih Tzu, and one each border collie, boxer, French bulldog, husky, Maltese, and whippet. The mean study population weight was 19.4 +/– 11.0 kg and median BCS 6/9 (range, 3.5–8), with no difference in weight between groups (standardized difference, 0.06 weight, 0.54 BCS). The median population age was 3.7 years (range, 1.5–14.4 years), with a median age of 2.7 years in FMT dogs (range, 1.5–11.0 years) and 8.5 years (range, 3.2–14.4 years) in probiotic dogs (standardized difference 1.12). Most dogs normally received a standard, commercial diet. Duration of clinical signs prior to presentation ranged from 4 to 48 h (median, 24 h). One FMT dog received a raw diet; this dog had no other signs consistent with acute Salmonellosis (e.g., fever, leukopenia). Five owners reported diet change (*n* = 2), introduction of new commercial treats (*n* = 2), or known dietary indiscretion (*n* = 1) within 1 week of clinical sign onset. Four dogs were receiving chronic medications (*n* = 1 oclacitinib, *n* = 1 lokivetmab, *n* = 1 meloxicam, *n* = 1 pimobendan) with no new medications or dose adjustments within the 3 months prior to presentation.

### 3.2. Clinicopathologic values

Table 1 lists selected clinicopathologic values for both groups. While there was no difference in baseline hematocrit (standardized difference 0.04) or band neutrophil count between groups (standardized difference 0.19), there was variation in baseline platelet count (standardized difference 0.27), leukocyte count (standardized difference 0.77), and segmented neutrophil count (standardized difference 0.69). Baseline serum albumin and total protein were lower in probiotic dogs vs. FMT dogs (standardized difference 1.38 albumin, 0.87 total protein).

**Table 1:** Selected baseline median (range) clinicopathologic values for 18 dogs with acute hemorrhagic diarrhea syndrome receiving either FMT or probiotic compared using standardized difference.

**Variable (Units)**	**FMT**	**Probiotic**	**Reference interval**	**Cohen's *d***
Hct %	60 (55–67)	63 (52–71)	41–59	0.04
Total leukocyte count x10^9^/L	10.7 (8.6–17.1)	9.3 (5.6–14.9)	4.3–13.6	0.77
Segmented neutrophil count x10^9^/L	9.2 (6.6–14.8)	8.5 (4.4–12.8)	2.5–9.3	0.69
Band neutrophil count x10^9^/L	0.0 (0.0–0.4)	0.0 (0.0–0.1)	0.0–0.1	0.19
Platelet count x10^9^/L/100x	275 (185–444)	260 (111–343)	130–370	0.27
Total protein g/dL	7.2 (6.4–8.0)	6.5 (5.4–7.8)	6.3–8.0	0.87
Albumin g/dL	3.9 (3.7–4.4)	3.7 (3.3–4.0)	3.2–4.2	1.38
Globulins g/dL	2.3 (2.1–3.0)	2.2 (1.3–3.1)	1.8–3.0	0.87

A Cohen's *d* > 0.25 indicated a baseline imbalance in the variable between dogs receiving FMT vs. probiotics.

FMT, fecal microbiota transplant.

### 3.3. Serum LPS

**Figure 2:**
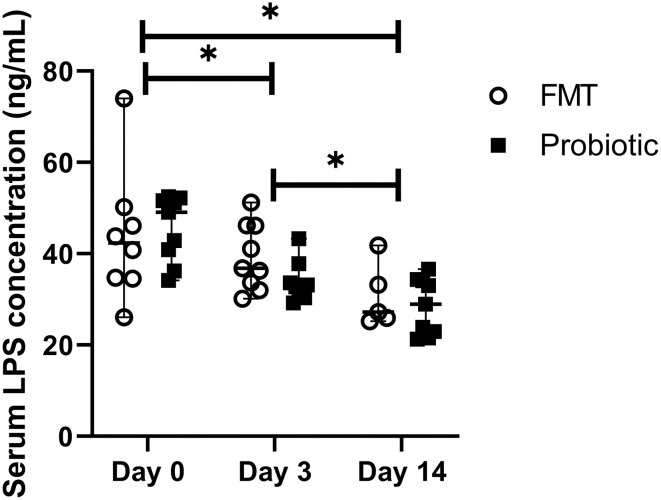
Line plot of serial serum lipopolysaccharide (LPS) concentrations in 18 dogs with acute hemorrhagic diarrhea syndrome receiving either fecal microbiota transplant (FMT) or probiotic. Data are expressed as median (range); data points represent individual dog values. Asterisk (*) indicates a significant difference (*p* < 0.05) when comparing baseline vs. days 3 and 14 and day 3 vs. day 14 in all dogs.

The average ELISA intra-assay CV was 11.2%, and the inter-assay CV was 12.1%. Baseline LPS did not differ between groups [FMT 42.3 ng/mL (range, 26.1–74.0 ng/mL); probiotic 49.1 ng/mL (range, 34.2–52.5 ng/mL); standardized difference 0.16]. LPS concentrations decreased over time, regardless of treatment group [baseline, 43.9 ng/mL (range, 26.1–74.0 ng/mL); day 3, 33.7 ng/mL (range, 29.2–51.2 ng/mL); day 14, 28.1 ng/mL (range, 21.3–41.8); *p* < 0.01; Figure 2]. LPS moderately correlated with fecal score (r_s_ = 0.65, 95% CI 0.45–0.79; *p* < 0.001) and disease severity score (r_s_ = 0.66, 95% CI 0.45–0.79; *p* < 0.001).

### 3.4. Fecal scores

**Figure 3:**
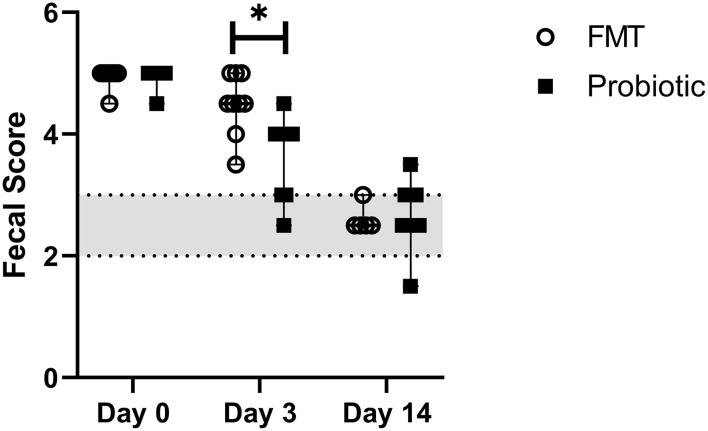
Line plot of serial fecal scores in 18 dogs with acute hemorrhagic diarrhea syndrome receiving either fecal microbiota transplant (FMT) or probiotic. Data are expressed as median (range); data points represent individual dog values. Asterisk (*) indicates a significant difference (*p* < 0.05) when comparing treatment groups on day 3. The shaded region denotes a normal fecal score, where a score ≥3.5 is considered diarrhea.

Median baseline fecal score was 5/5 (range, 4.5–5.0) in both groups and decreased over time, with lower scores on days 3 (4.0; range, 2.5–5.0) and 14 (2.5; range, 1.5–2.0) vs. baseline and lower scores on day 14 vs. day 3 (*p* < 0.001). Day 3 fecal score in probiotic dogs 3.67 +/– 0.66 was lower than in FMT dogs 4.50 +/– 0.50 (*p* = 0.03; Figure 3). Mean time to fecal score 3 was 7.17 +/– 3.06 days in FMT dogs vs. 5.00 +/– 2.29 days in probiotic dogs (*p* = 0.14).

### 3.5. Disease severity scores

**Figure 4:**
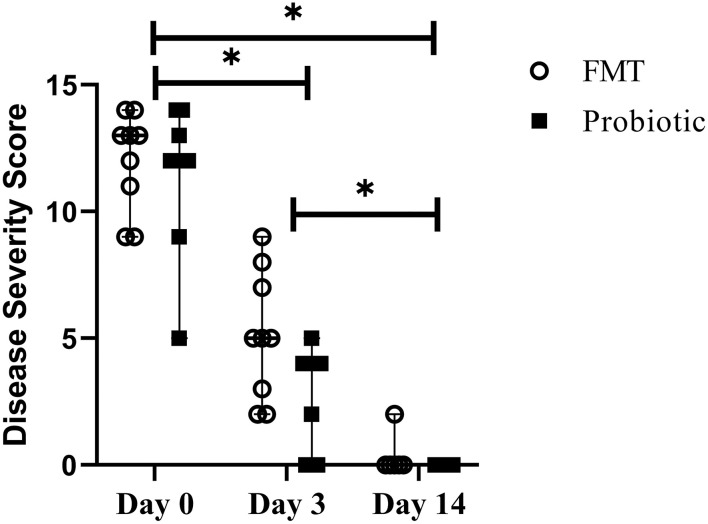
Line plot of serial disease severity scores from 18 dogs with acute hemorrhagic diarrhea syndrome receiving either fecal microbiota transplant (FMT) or probiotic. Data are presented as median (range); data points represent individual dog values. Asterisk (*) indicates a significant difference (*p* < 0.05) when comparing baseline vs. days 3 and 14 and day 3 vs. day 14 in all dogs.

There was no difference in baseline disease severity score between FMT dogs [median 13 out of 15 (range, 9–14)] and probiotic dogs [median 12 (range, 5–14); Standardized difference 0.23]. Disease severity score decreased over time in both groups and was lower on days 3 [median 4 (range, 0–9)] and 14 [median 0 (range, 0–2)] vs. baseline and day 14 vs. day 3 (*p* < 0.001). There was no difference between groups over time (*p* = 0.05; Figure 4).

### 3.6. Adverse effects and study outcomes

Although there was no specific adverse effect monitoring performed, no obvious adverse effects secondary to probiotic administration were noted. FMT dogs commonly displayed self-limiting ptyalism during the procedure, which resolved following enema completion. All dogs, except the two FMT dogs that were discharged prior to the recommendation (later), began eating the standardized diet within 24 h of hospitalization, and the percent intake gradually improved. No appetite stimulants or enteral feeding (e.g., nasogastric tube) were used.

Fourteen of 18 enrolled dogs completed the study (*n* = 9 probiotics; *n* = 5 FMT; *p* = 0.08; 95% confidence interval 1.09–8.68). All 18 dogs were included in day 3 analyses and 14 dogs on day 14. The median days to discharge was 3 (range, 2–4) for FMT dogs and 2 (range, 1–3) for probiotic dogs (*p* = 0.09).

While no dogs were euthanized as part of the study, two FMT dogs were euthanized on days 4 and 7, respectively. While both dogs initially improved, owners declined continued hospitalization on day 3 due to financial constraints. At the time of discharge, vomiting had resolved in both dogs, but the fecal score was 5, defecation frequency was 4–5 times per day, and dogs were eating ≤ 50% RER. One dog was re-presented on day 4 for inappetence and weakness. It was euglycemic (7.1 mmol/L), with stable Hct from discharge (46%). The owners elected humane euthanasia, and necropsy demonstrated necrohemorrhagic, fibrinoulcerative enteritis with intralesional bacilli, fibrinosuppurative pneumonia with intralesional cocci, and pulmonary vasculitis. PCR on GI tissue was negative for Salmonella, canine enteric coronavirus, canine parvovirus 2, canine distemper virus, *C. difficile* toxins A and B, and *Lawsonia intracellularis* and positive for *C. perfringens* alpha toxin gene. The second dog re-presented on day 5 and demonstrated fever (40.6°C), pitting edema of all limbs, and hematologic evidence of systemic inflammation and consumptive coagulopathy; it was withdrawn from the study at this time. Abdominal ultrasound findings were consistent with non-specific gastroenteritis, and humane euthanasia was elected on day 7.

Two additional FMT dogs were withdrawn on days 4 and 10. One was withdrawn due to continued diarrhea following discharge on day 3, owner dissatisfaction with the clinical improvement rate, and subsequent metronidazole prescription by the dog's primary veterinarian. The last dog was withdrawn due to amoxicillin administration for a urinary tract infection diagnosed based on clinical lower urinary tract signs and positive urine culture.

## 4. Discussion

This prospective study evaluated an oral multi-strain probiotic vs. enema-administered FMT for the treatment of non-septic canine AHDS, with the goal of comparing two microbiome-targeted therapies on clinical response and serum LPS, as a marker of GI permeability. In this population, both treatments were well-tolerated during administration, and LPS concentrations correlated with fecal scores and disease severity scores.

Probiotic and FMT impact on canine AHDS were preliminarily evaluated in separate populations, in one study each (6, 25). Those studies suggested possible benefits on the GI microbiome (6), metabolome (25), and disease duration (6). Similar to those studies, fecal consistency and disease severity improved rapidly in most dogs in this study. Using the same probiotic and a similar dose, the day 3 disease severity score of 4/15 observed in this population is similar to that observed by Ziese et al. (5/18), denoting mild disease (6). Differences in study design preclude direct comparison of the probiotic impact on fecal characteristics. Improvement in the fecal microbiome dysbiosis index was noted in AHDS dogs receiving probiotics, but the improvement was comparable to placebo, and fecal scoring was not performed (6).

Although population size limited statistical significance, time to fecal score normalization and disease duration based on severity scores were higher in FMT vs. probiotic dogs. Furthermore, the day 3 fecal score was higher in FMT dogs and more probiotic dogs completed the study, with three FMT dogs withdrawn due to progressive disease or owner dissatisfaction with the improvement rate. Study completion was unlikely related to differences in disease severity, as there were no differences in biomarkers of GI permeability or standardized severity scores at baseline. While there were minor biochemical differences between groups based on standardized differences, these were considered clinically insignificant. Had the three withdrawn dogs completed the study, differences between groups in the rate of improvement in those parameters might have been further emphasized. Although positive metabolome effects were previously reported following FMT (25), differences in clinical responses (i.e., day 3 fecal score) to probiotics and FMT in this study highlight the importance of evaluating new therapies in one population.

Two FMT dogs were euthanized during the study. While the necropsy was only performed in one dog, no concurrent diseases were identified, making death likely due to AHDS complications. One dog had GI transmucosal bacterial involvement on histopathology, as well as evidence of hematogenous pneumonia. However, mucosal bacterial involvement is consistent with previous findings in AHDS dogs (4). Both dogs were discharged prior to the recommendation, reinforcing the importance of in-hospital care until adequate clinical improvement occurs. As some AHDS dogs may develop life-threatening complications from bacterial translocation, future studies should evaluate which dogs would benefit from antimicrobials. While FMT administration appeared well-tolerated, detrimental effects cannot be excluded. In humans, FMT carries a low risk of sepsis with appropriate donor screening (39); though, septic complications have been reported (40, 41). Fecal donors in this study were screened for select enteropathogens. In addition, euthanized dogs received FMT material from different donors, and other dogs received FMT material from those donors without complication. While direct enteropathogen transmission through FMT was considered unlikely, the potential risk of sepsis highlights the need for standardized infectious disease screening for FMT donors and assessment of risk factors for FMT complications in AHDS dogs.

Decreased GI barrier function has been demonstrated in canine AHDS based on GI protein loss (2) and histopathological findings of GI bacterial translocation (4). While increased fecal α-proteinase inhibitor demonstrates hyperpermeability, serum LPS could be a more specific marker of bacterial translocation. In humans with sepsis, serum LPS increases, regardless of blood culture positivity (42, 43). LPS increases in naturally occurring (42–46) and induced (47) GI hyperpermeability models or decreased systemic bacterial clearance (48) but has not been previously evaluated in AHDS dogs. While other GI permeability biomarkers have not correlated with AHDS outcomes (2), LPS correlated moderately with fecal scores and disease severity. Furthermore, LPS decreased over time in both groups, suggesting initial GI hyperpermeability, which improved with disease resolution. These findings make LPS an intriguing potential biomarker for AHDS disease severity and recovery. Dogs in this study had detectable serum LPS at study completion, which contrasts with undetectable concentrations observed in healthy dogs in one study (44). However, another study that induced GI hyperpermeability also detected LPS in healthy dog serum (49). Continued monitoring for LPS decrease and LPS quantification in healthy dogs with the method used in this study would be needed to assess the resolution of GI hyperpermeability.

There are several population and study design criteria important to consider in relation to this study's results. Dogs in the FMT group were younger than the probiotic group. As dogs were randomized to the treatment group based on sequential presentation, this is likely due to random chance. The median age of 2.67 years in FMT dogs is more similar to other canine AHDS studies (2, 7, 9) than the median of 8.5 years in probiotic dogs; however, dogs up to 16 years of age are affected (7, 25, 36). To the authors' knowledge, the effect of age on response to supportive AHDS treatment has not been reported. It is possible that disease could differentially impact the microbiome of younger dogs. There are substantial shifts in GI bacterial microbiota in developing puppies (50), but broad population analyses suggest relatively stable microbiome compositions between young adults and older individuals (51, 52). Microbiome analysis would be required to evaluate the influence of age on the severity of dysbiosis at disease onset or clinical recovery. The Hct cut-off chosen for inclusion was arbitrary and falls within the reference interval for many diagnostic laboratories. However, there is no accepted Hct cut-off for the diagnosis of AHDS. Most published veterinary studies do not state Hct as an inclusion criterion, (2, 4, 6, 7, 9, 10, 36) and a range from 33 to 76% has been reported, (36) suggesting that the degree of hemoconcentration is less important than clinical disease presentation. The study inclusion time-frame of 48 h was also shorter than, though falls within, the < 3 days used by many studies. (8, 10, 19, 36) However, disease duration is not defined in all previous studies (5, 9), and only three dogs in the current study presented at the 48-h duration of clinical signs allowed, suggesting that dogs often present to veterinarians well within 72 h due to the severity of clinical signs. Several dogs in this study also had known dietary indiscretions or recent introduction of novel commercial dog foods or treats. Although AHDS is considered an idiopathic condition, dietary factors are considered a possible trigger (53). Despite this, dietary factors are uncommonly reported in literature describing AHDS and warrant further consideration in future studies. Abdominal imaging and additional blood work (e.g., baseline cortisol) were at the discretion of attending clinicians based on individual patient presentation. While diseases such as pancreatitis and hypoadrenocorticism can have similar disease presentations, the rapid recovery and normal clinical status at recheck made these diseases unlikely. In the two dogs that did not respond to therapy, abdominal ultrasound and necropsy did not find other disease etiologies. While a lack of imaging could be considered a limitation, this is consistent with previous veterinary literature (5, 8, 9). These study variabilities highlight the substantial differences in veterinary literature among AHDS studies, making comparisons among studies challenging, and highlighting the need for standardized veterinary studies to accurately compare treatment efficacy.

This study had several important limitations. Fecal microbiome analysis was not performed in either the study population or FMT donors. This limits assessment in the study population to clinical signs and indirect measures of treatment effect (e.g., fecal scoring). However, fecal scores are repeatable when performed by a single observer (54). While microbiome evaluation at different time points could help direct adjustments that could impact efficacy (e.g., need for repeated FMT), the use of fecal scores and disease severity scores to define AHDS improvement is consistent with available literature (2, 19, 22, 25). In addition, while FMT improved fecal metabolite profiles in AHDS dogs (25), that study also failed to demonstrate clinical benefit. In the context of canine AHDS, clinical benefits, such as the rate of diarrhea improvement, are arguably of greater importance given that clinical scores directly reflect patient morbidity and treatment burden. Fecal microbiome and metabolome analyses in FMT donors would have been useful to demonstrate that the material used for FMT contained beneficial bacterial populations and metabolic by-products. This should be a consideration for future studies, particularly given the lack of clinical improvement noted both in this study and previous studies in AHDS dogs (38) following FMT. Given the uncommon potential for the transmission of pathogenic, antimicrobial-resistant microbiota (40), microbiome analyses should be considered for both the safety and efficacy screening of FMT products. This study also selected a patient population with moderately severe disease. The dogs were sick enough to require hospitalization but recovered without the need for nutritional support. Therefore, it is unknown if the findings of this study can be applied to dogs with different severities of clinical signs. Furthermore, the number of dogs presenting through the emergency service who were not considered for enrollment by admitting clinicians (i.e., the study team was not notified for enrollment screening), either due to owners declining hospitalization or biochemical parameters suggesting severe (e.g., hypoglycemia, neutropenia) or systemic disease is unknown. Therefore, the likelihood of AHDS vs. other diseases in dogs with this clinical presentation cannot be determined from this study. Finally, independent full validation of the ELISA used in this study was not performed. Given the differences in circulating LPS concentrations noted in studies using varying measurement techniques (44, 49) and the lack of available commercial gold standard tests, this is an important consideration for future studies when comparing results to other laboratories or patient populations.

Microbiome-targeted treatments are not interchangeable; this includes probiotic type, dose, and duration, (18) as well as FMT method or timing. While human studies have not consistently demonstrated a difference among FMT administration methods (55–59), this might differ in dogs in which ensuring retention time is challenging. In our study, dogs were not walked or fed for 4 h post-enema, but some dogs defecated shortly after FMT. The above-mentioned study (25) utilized colonoscopy to prolong retention but also noted no short-term clinical benefit. Other methods to prolong retention or repeated administration might improve efficacy. We elected not to perform sedation or anesthesia for FMT administration due to the severity of the patients' illness and ethical considerations to avoid procedures that might induce hypotension and further decrease GI perfusion. Ideally, all dogs would have received FMT material from one donor. Given the prospective study design and microbiome stability in frozen samples (30, 59), this was not feasible. Dogs could not serve as repeat donors due to exclusion criteria (e.g., perioperative antimicrobial administration) following the first donation. This reflects normal limitations in fecal donation that occur in clinical practice. These limitations highlight the need for standardized studies evaluating microbiome-targeted therapies and assessment methods to accurately compare treatments.

Small study sizes likely impacted the ability to determine a significant difference between treatment groups. *Post-hoc* power analysis comparing average days to achieve a fecal score of 3, demonstrated that 27 dogs per group would be needed. We speculated that differences between groups would have been highlighted had several FMT dogs not withdrawn, as those dogs continued to have high severity scores and fecal scores at the time of withdrawal and were not included in outcome measures. A larger study cohort will allow the assessment of whether the trends in the normalization of fecal scores, improvement in disease severity scores, and days to discharge become statistically significant. The inclusion of additional dogs will also provide further evidence for tolerance of these therapies or potential side effects (e.g., septic complications). Most dogs were not maintained on a single diet throughout the course of the study, as recommended but rather were transitioned back to their normal diets by owners. While diet affects the GI microbiome (60–62), diets were hugely variable across both groups, making it an unlikely contributor to the observed treatment effects. Finally, this study lacked an untreated (i.e., supportive care only) control group, which precludes the assessment of whether probiotics or FMT improves disease course or could result in detrimental effects compared to supportive care alone. Multiple studies have demonstrated the effectiveness of supportive care (e.g., IV fluids, anti-emetics) for the treatment of AHDS, without the need for ancillary, GI-directed therapies. (2, 6, 8, 19, 36) Although the duration of hospitalization is generally short, this can be cost-prohibitive for some owners; therefore, therapies with the potential to decrease the duration of clinical signs and hospitalization would still be of benefit. Practitioners are exploring both probiotics and FMT as the treatment for AHDS, and studies in independent populations have demonstrated clinical (6) or microbiome benefits (6, 25) when compared to supportive care. As probiotics and FMT have not been evaluated in a single population, the direct comparison provides a valuable clinical perspective. However, when considering the need to provide either therapy to AHDS dogs, future studies should include an untreated control group to determine whether they provide clinical benefit.

In this prospective, single-site trial, there was no difference in the clinical disease course in AHDS dogs receiving a single enema-administered FMT in the initial treatment period compared to probiotic supplementation at a dose of 200 CFU/10 kg; though, the small study population limited statistical ability to detect a meaningful difference. Association of LPS with fecal consistency and disease severity warrants further evaluation of LPS as a biomarker of disease severity and recovery. Future studies should evaluate different FMT administration methods in AHDS dogs and directly compare microbiome and metabolome impact, as well as clinical significance in an untreated control group.

## Data availability statement

The raw data supporting the conclusions of this article will be made available by the authors, without undue reservation.

## Ethics statement

The animal study was reviewed and approved by Kansas State University Institutional Animal Care and Use Committee. Written informed consent was obtained from the owners for the participation of their animals in this study.

## Author contributions

MJ was responsible for the development of the study idea, study design, data collection and analysis, and manuscript preparation/review. KK was involved in study design, data collection, and manuscript preparation/review. LF was involved in data collection and manuscript preparation/review. All authors contributed to the manuscript and approved the submitted version.
